# The Stockholm Follow-up Study of Users Diagnosed with Psychosis (SUPP): A 10-year Follow-up 2004–2013

**DOI:** 10.1007/s10597-020-00740-2

**Published:** 2020-11-15

**Authors:** Per Bülow, Alain Topor, Gunnel Andersson, Anne Denhov, Claes-Göran Stefansson

**Affiliations:** 1 Department of Social Work, School of Health and Welfare, Jönköping University, Jönköping, Sweden; 2 Regional Forensic Psychiatric Hospital, Vadstena, Sweden; 3 Department of Psychosocial Health, University of Agder, Kristiansand, Norway; 4 Department of Social Work, Stockholm University, Stockholm, Sweden; 5 Stockholm, Sweden

**Keywords:** Severe mental illness, Longitudinal study, De-institutionalisation, Psychiatric care, Social services

## Abstract

Since the 1970s, psychiatric care in the western world has undergone fundamental changes known as de-institutionalisation. This has changed the living conditions for people with severe mental illness. The purpose of this study was to investigate the living conditions and utilisation of care and social services for a group of people in Sweden with diagnosis of psychosis over a 10-year period, 2004–2013. During this period, psychiatric care decreased at the same time as interventions from the social services increased. Half of the persons in the studied group did not have any institutional care, that is, neither been hospitalised nor dwelling in supported housing, during the last 5 years, and just over 20% had no contact with either psychiatry or the municipality's social services during the last 2 years of the investigated period.

## Background

Psychiatric care has undergone major changes in the western World since the mid-20th century, often referred to as de-institutionalisation. In its simplest form, de-institutionalisation means that the old mental hospitals were downsized and replaced by psychiatric out-patient units and community-based care, performed outside the large-sized institutions (Tansella and Thornicroft 2001).

However, the concepts of de-institutionalisation and community-based care are multifaceted, and the organisational changes have created a complex landscape of psychiatric “supplies” (Topor et al. 2015). De-institutionalisation and the expanding community-based care have different meanings and are interpreted in divergent ways. Moreover, there are major differences between countries regarding when the de-institutionalisation process started and the pace of the subsequent ongoing process (Goodwin 1997). In Sweden, the process began in the 1970s with a nationwide implementation of Community Mental Health centres with out-patient units as a complement to psychiatric hospitalisation. Every psychiatric clinic should be responsible for all in- and out-patient treatment in a defined catchment area (Sectorisation). Evaluations showed that patients with severe mental illness (SMI) did not benefit from out-patient care due to lack of support in social needs relating to daily life matters. As a response, the Swedish Government commissioned a parliamentary committee to make proposals for a reformation of psychiatry in Sweden. It resulted in The Mental Health Care Reform, which came into being in 1995.

The Mental Health Care Reform clarified the responsibilities of social services and psychiatry respectively, and approximately 15% of the county council-controlled budget for mental health care was transferred to the municipal social services. The social services responsibility was to provide support to persons with SMI in housing, employment and an active everyday life, thereby creating conditions to integrate into society. The task of the county council psychiatry was to develop psychiatric treatment and to treat and prevent SMI. The reform emphasised that social services and psychiatry must cooperate to prevent people from “falling between the cracks”.

The transfer of psychiatric care from mental hospitals to community-based care proved to be more complicated than predicted. Previously, when people with SMI lived their lives in mental hospitals, their vital needs were taken care of by one and the same organisation. In de-institutionalised and community-based care and support, responsibility was divided between different organisations. This created, to a certain extent, boundary issues about who had the responsibility between the different organisations. This meant that new problems arose. Earlier follow-up studies reported that people who previously lived in mental hospitals became homeless (Craig 1998), ended up in prisons (Modestin and Ammann 1995), died prematurely (Mortensen and Juel 1993) and committed suicide to a greater extent (Taylor and Gunn 1999). Thus, de-institutionalisation was criticised, especially for lack of alternative community-based care (Warner 1998). Munk-Jørgensen (1999) noted a wide range of problems and wondered if the de-institutionalisation had not gone too far.

The use of long-term follow-up studies is an important research strategy to increase knowledge about care and living conditions for people with SMI. Long-term follow-ups are, however, a challenge because it is difficult to trace and follow people over a longer time, especially in countries that do not have personal identity numbers linked to population registers. There are epoch-making studies which, despite being carried out decades ago, are still influential (Ciompi 1980; Harding et al. 1987). They challenged the prevailing notion that schizophrenia was an incurable disease by presenting numbers that showed that even the “worst cases” recovered. In a later study, Harrow et al. (2005) found in a 15-year follow-up that over 50% of patients with a schizophrenia diagnosis did not have a chronic course. Rather, the illness was episodic with periods of recovery.

More recent published longitudinal studies have followed the clinical course and outcome of psychosis (Kotov et al. 2017), trajectories of social functioning (Velthorst et al. 2017) and quality of life and social functioning (McInerney et al. 2018). Results from the studies by Kotov et al. and Velthorst et al. pointed to a gradual deterioration in both symptoms and social functions, but as Kotov (2017) pointed out, it might be a result of inadequate care and support efforts. The results from the study by McInerney et al. displayed a contrary result. A group of long-stay patients, the majority of whom had a diagnosis of schizophrenia, were followed over a period of 10 years after being discharged from a mental hospital and reported long-term improvements in both quality of life and social functioning.

A study by Lora et al. (2011) emphasised that insufficient economic resources hinder the intention of de-institutionalisation, which is to improve the living conditions for persons with SMI and facilitate the transformation from a patient to a person, and to be a citizen in the society. To achieve the purpose, Lora et al. emphasise that community-based care must be prioritised. However, irrespective of how psychiatric care and social services are organised, people with SMI face multiple challenges regarding employment, income, accommodations, and psychiatric and physical health (Morgan et al. 2012).

Despite the uncertainty about how the de-institutionalisation process exerts influence on the outcome of people with SMI, this process continues with reduced hospital beds and increasing community-based care, according to Chow and Priebe (2016). However, Chow and Priebe raise the question of whether re-institutionalisation has replaced de-institutionalisation, i.e. the number of beds in mental hospitals has been replaced by beds in forensic psychiatry, accommodation in supported housing or an increasing prison population. The British psychiatrist Lionel Penrose launched a hypothesis in 1939 that the number of hospital beds and the number of people in prison was in direct relation to each other. This hypothesis was updated in the mid-2000s (Priebe et al. 2005). Since then, the interest in studying the relationship between care facilities and the number of persons in prison has been ignited (Mundt et al. 2015; Blüml et al. 2015).

The divergent results indicate the need to have follow-up studies addressing the question: what are the living conditions and care situation for people with SMI and how do they change over time?

The present study is a continuation of a previously published study "The Stockholm Follow-up Study of Users Diagnosed with Psychosis (SUPP): Methodology, Patient Cohort and Services" (Topor et al. 2012) studying living conditions and utilisation of health care and social support for the surveyed population during the period 1997–2004. This study follows the same cohort, over the 10-year period from 2004 to 2013.

The overall purpose of this study is to map out and describe living conditions and utilisation of health care and social support for the surveyed population over a 10-year period. The findings are presented and analysed from a gender perspective.

## Data and Method

The study is a longitudinal and naturalistic cohort study. All patients with a diagnosis of psychosis, according to the inclusion criteria, were included, and data presented reflected a psychiatric organisation that offered psychiatric care according to treatment as usual (TaU). Data were retrieved from central registers: Statistics Sweden (SCB), National Board of Health (including Cause of Death Register and National Inpatient Register), The Swedish National Council for Crime Prevention, and Regional databases from social services and county council. As this study is a continuation of a 4-year follow-up of the same survey population, a more detailed report of the data is presented in Topor et al. (2012).

### Population Sample

The study comprises 1501 people who had contact with a psychiatric sector organisation in Stockholm South in Sweden. The inclusion criteria were persons registered in the local hospital base during the period 1997–2004 and who had a psychosis diagnosis within the F20–F29 classification group, according to ICD-10 and had contact with psychiatry in Stockholm in 2004. Organic diseases such as dementia and brain damage that caused psychotic symptoms were excluded as well as persons with protected identity. Twenty-one people declined participation; thus, we present data pertaining to 1480 people.

During the 10-year period, 254 people died, evenly distributed between the sexes: 127 women and 127 men. Mean age at death was 68 years for women and 60 years for men. This can be compared to the life expectancy in Sweden in 2015, which was 84 for women and 80 for men (SCB 2016). The most common causes of death were diseases of the circulatory system and tumour diseases, which are the most common causes of death among the Swedish population. The proportion of suicide in the studied population was high, especially among men. The proportion of suicide, both sexes, is 10 times higher compared to the general population.

In the following text, only people who were still alive in 2013 are included, consisting of 1226 people. However, 10 persons are missing in all registers, which means that we have data from 1216 persons: 597 women and 619 men. However, due to shortcomings in reporting data, meaning that the data is not always complete in all registers, the total sum varies in Table 2.

### Setting

At baseline, in 2004, all of the patients in this study had contact with Psychiatry (South) in Stockholm, which was a sector clinic with a specified area covering 232,000 inhabitants over 18 years old. In addition to general psychiatric care, the clinic also offered specialised care for persons with diagnosis of psychosis, both in- and out-patient care. There were no private actors that provided psychiatric care to persons with SMI during the time of the investigation.

### Age and Sex

The studied population was evenly distributed between the sexes: 729 (49.3%) women and 751 (50.7%) men. Age ranged from 18 to 93 years, with a median age of 47 years (men 45 years and women 50 years) at the beginning of 2004  (Table 1).

**Table 1:** Age distributed between the sexes in the studied population, number of persons and percentage in (%)

Age	Men	Women	Total
Below 24	28 (5)	22 (4)	50 (4)
25–34	104 (17)	83 (14)	187 (15)
35–44	192 (31)	161 (27)	353 (29)
45–54	199 (32)	173 (29)	372 (31)
55–64	76 (12)	108 (18)	184 (15)
65–74	15 (2)	30 (5)	45 (4)
75 and over	5 (1)	20 (3)	25 (2)
Total	619 (100)	597 (100)	1216 (100)

Several of the persons in this study have had a long history of SMI and contact with psychiatric care. At baseline, in 2004, 45 persons (3% of the studied population) had their first contact with psychiatry; however, 40% (n = 501) had their first contact 16 years or further back in time in relation to baseline in 2004. In 2004, 152 persons were 65 years or older; in 2013, the figure was 254 persons: 96 men and 158 women. In Sweden, the retirement age is 65 and implies changes in income and housing conditions.

## Results

Demographic data refer to vital living conditions, including information on marital status, education, housing and disposal income. In the section psychiatric and social interventions, we present data on both in- and out-patient care provided by the county council and interventions from social services.

### Demographic and Economic Data

There are small changes in the survey group regarding marital status, education, housing and economic status during the studied period 2004–2013, see Table 2.

**Table 2:** Demographic data, number of persons, percentage (%) in the years 2004 and 2013 and p-value for differences between men and women in 2013

	Men		Women		P-value
	2004	2013	2004	2013	
Marital status					
Unmarried	470 (76)	444 (73)	357 (60)	325 (55)	<.005
Married	61 (10)	57 (9)	74 (13)	70 (12)	ns
Divorced	85 (14)	104 (17)	138 (23)	158 (27)	<.005
Widower	1 (0)	4 (1)	26 (4)	36 (6)	<.005
Total	617 (100)	609 (100)	595 (100)	589 (100)	
Education					
9 year compulsory school	164 (27)	165 (27)	122 (21)	126 (22)	<.005
Upper secondary school	294 (49)	289 (48)	258 (46)	263 (45)	ns
Post upper secondary school	142 (24)	147 (25)	184 (33)	191 (33)	<.005
Total	600 (100)	601 (100)	564 (100)	580 (100)	
Housing					
Self-contained houses	12 (2)	24 (4)	22 (4)	40 (7)	ns
Rental flats	563 (94)	501 (87)	557 (95)	508 (89)	ns
Other	25 (4)	53 (9)	7 (1)	22 (4)	<.005
Total	600 (100)	578 (100)	586 (100)	570 (100)	
Disposal income per year^*^					
1–120,000	425 (68)	356 (58)	358 (60)	273 (46)	<.005
120,001–240,000	180 (29)	237 (38)	227 (38)	286 (48)	<.005
241,001–360,000	10 (2)	19 (3)	9 (2)	28 (4)	ns
360,001-	4 (1)	7 (1)	1 (-)	10 (2)	ns
Total	619 (100)	619 (100)	597 (100)	597 (100)	

^*^100 Swedish Crowns (SEK) is equivalent to approximately 10 EUR, 12 USD or 8 GBP

#### Marital Status

Some 10% of the men and 12% of the women were married over the period. This figure is about one-third compared to the general population in Sweden, where about 33% of the population were married during the same time. The marital status did not change significantly, and the changes that occurred during the 10-year period were an increase in the proportion of divorce and the proportion of those who became a widow/widower. These changes can probably be explained by increasing age.

#### Education

The level of education was stable during the studied period, and few persons continued to study after 2004. Compared to the general population in Sweden, fewer persons in our studied group (29%) had post upper-secondary education; the corresponding figure for the Swedish population was 43%.

#### Housing

A majority lived in flats, which is most common in this area of Stockholm, throughout the period. In 2004, 6% were residing in supported housing, distributed between men 8% and women 5%. In 2013, the figure had increased to 13% for both sexes. Homelessness is rare in Sweden, especially in groups with SMI (The National Board of Health and Welfare 2017).

#### Disposal Income

The disposal income was based on persons younger than 65 and were thus in working age. The proportion of women and men who had some form of employment income decreased between 2004 and 2013. The number of women declined from 193 (32%) in 2004 to 133 (22%) in 2013. The corresponding figures for men were 161 (26%) in 2004 and 125 (20%) in 2013. The most common source of income was early retirement pension due to disability or sickness. This was the fact for 61% of the women and 66% of the men in 2004, with few changes during the 10-year period. Other sources of income were old age pension, sickness benefit, salary and social allowance. The most salient change during the 10-year period was in social allowance, which declined with 55% for the women and with 63% for the men.

The disposable income, in median value, increased slightly during the 10-year period, from 114,000 to 121,000 SEK for women and from 110,000 to 115,000 for men. By comparison, the average disposable income in Stockholm was more than threefold in 2013.

### Psychiatric Care and Social Interventions

Psychiatric care is defined in this paper as in-patient care at psychiatric clinics and out-patient care by psychiatric teams. All psychiatric in-patient care in Sweden was provided by the county council at that time. This holds for out-patient care as well but it can be accomplished by private actors, although they are in a minority. Out-patient care could either be performed at the patient’s home, by home-visiting teams or, more commonly, at the psychiatric out-patient care unit where the patient visits a nurse, psychologist and/or a psychiatrist after an appointment.

Social interventions are defined as support in daily living, including living in supported housing, home help service, occupational support and financial support. Other interventions include transportation service, companion service, etc. The social interventions are provided by teams specialised in working with persons with SMI arranged by the municipalities.

#### Utilisation of Psychiatric In- and Out-patient Care

During the 10-year period, both in- and out-patient care were reduced. Admittance to psychiatric clinics decreased in the first 4 years 2004–2007, and then remained at a level where 15–18% of the studied population have at least one period of in-patient care per year.

A majority of the persons that were hospitalised had short periods of treatment. On average, each year, 9% of the persons that were hospitalised had treatment durations between 3 and 6 months per year, 5% between 7 and 11 months and 2% the whole year. Being hospitalised the whole year was three times more common among men compared to women; otherwise, there were no differences between the sexes. These percentages did not differ during the 10 years investigated.

**Fig. 1:**
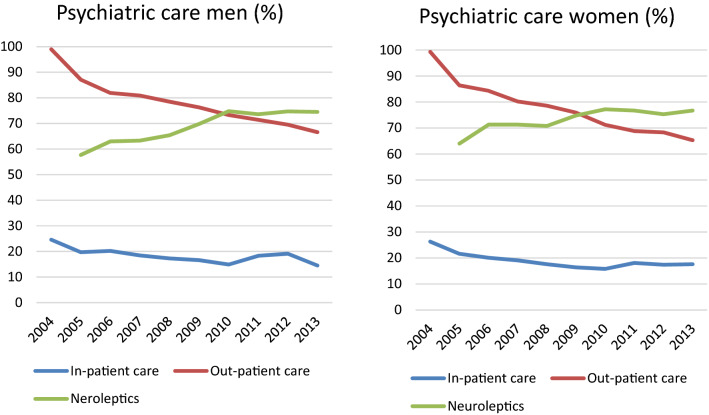
Out- and in-patient care and prescription of neuroleptics, the year 2004–2013. Neuroleptics is defined as ATC-codes N05A, excluding lithium (N05AN)

All forms of psychiatric, both in- and out-patient, care decreased continuously as seen in Fig. 1. Most notable is the decrease in out-patient care, where the visits were reduced by some 30%. There were no differences between the sexes. There were no major organisational changes in psychiatric care in the region that can affect the numbers in the period 2004–2013.

#### Prescription of Neuroleptics

Drug treatment is a cornerstone in the treatment for psychosis, and Swedish national guidelines advocate for treatment using neuroleptics in cases of psychosis diagnosis. The national register for medication started in 2005; consequently, there are no data for 2004. The number of persons prescribed with neuroleptics increased from 62% in 2005 to 77% in 2010 and remained at this level.

#### Social Services

There are a number of social services interventions available for persons with physical and psychiatric disabilities. Social services are governed by the Social Service Act, and include the most comprehensive of supported housing accommodation, where staff is available 24 h a day. Other interventions include, for example, mobility service, food delivery, companion, personal assistants and emergency medical alarm. In this article, we also address financial support as an important intervention for people with SMI. Poverty is often related to severe psychiatric problems and has a direct impact on the person’s well-being and psychiatric symptoms. In 2004, financial support was an important source of income, and 20% of the investigated population had such support at that time. Ten years later, in 2013, this figure had decreased to 7%, which may be due to the fact that more persons reached the age of 65 years and then received retirement pension. Over the 10-year period, more men than women had financial support; however, in 2013, the figure was 7% for both sexes.

**Fig. 2:**
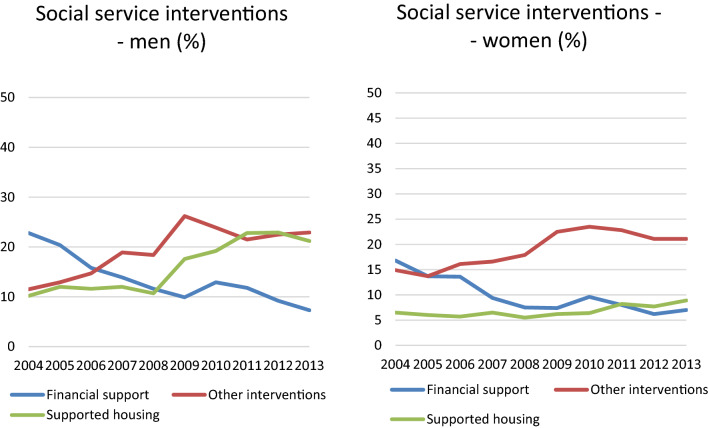
Social interventions

Residing in supported housing increased, especially among men. Nearly twice as many men lived in supported housing compared to women in 2013. Supported housing and employment support decreased, while home services remained at the same level (Fig. 2).

### Probation Services and Forensic Psychiatry

A constantly relevant issue is the relation between SMI and crime and if reduced in-patient care implies that people with SMI have instead been sentenced to imprisonment or to forensic psychiatry. According to Swedish law, no person may be sentenced to jail if the crime was committed under the influence of a serious mental disorder, and the court is obliged to sentence the person to care in forensic psychiatry. The crime should in those cases be of a kind that prison would be a "normal" sanction (Table 3).

**Table 3:** Sanctions for crimes

Year	Imprisonment			Forensic psychiatry		
	Men	Women	Total	Men	Women	Total
2004	4	–	4	6	–	6
2005	5	–	5	4	–	4
2006	3	–	3	4	1	5
2007	2	–	2	1	–	1
2008	3	1	4	3	–	3
2009	2	1	3	1	–	1
2010	-	–	–	1	–	1
2011	4	1	5	1	2	3
2012	2	–	2	1	–	1
2013	2	–	2	3	–	3

There are small differences in the number of sanctions during the investigated 10-year period. In total, 18 people have been sentenced to jail: 17 men and 1 woman. Nine persons have more than one prison sentence during this period. During the period, 27 people were sentenced to forensic psychiatry: 24 men and three women. One person has been sentenced to forensic psychiatry twice and two people have been sentenced to both prison and forensic psychiatry. In all, 43 persons, 39 men and 4 women, have been sentenced to prison and/or forensic psychiatry, which represents 3.5% of the studied population.

### How Many Have No Contact With Either Psychiatric Care or Social Services?

In this study, we investigate how many people who have an ongoing need, provided by the county council psychiatry and/or municipal social services, or the reverse, have no care or contact at all, as shown in Table 4. We chose to measure how many of the follow-up population who had no periods of institutional care during the last five and two years of the studied 10-year period. In the concept of institutional care, we include in-patient care at psychiatric clinics and living in supported housing. We also have the same cut-off periods regarding how many that had no interventions at all.

**Table 4:** Figures and number of persons with no institutional care and no interventions

	Men (%)		Women (%)		Total (%)	
	5-year	2-year	5-year	2-year	5-year	2-year
No institutional care	296 (48)	362 (58)	329 (55)	400 (67)	625 (51)	762 (63)
No intervention	79 (13)	124 (20)	89 (15)	147 (25)	166 (14)	271 (22)

As seen in Table 4, a majority of the follow-up population had no periods of institutional care during the last five (51%) and two years (63%), respectively. When we looked at the figures regarding no interventions at all, we found that 14% had no interventions during the last five years of the 10-year period and 22% the last two years. With no interventions, we mean no periods of in-patient care, contact with psychiatric open-care units or interventions from social services. More women than men had periods without institutional care, but when it comes to no interventions at all, the difference between the sexes decreases.

## Discussion

The purpose of this study was to investigate the living conditions and utilisation of care and social services for a group of people in Sweden with a diagnosis of psychosis over a 10-year period, 2004–2013. Although the purpose is not to test the Penrose hypothesis from 1939 of an inverse relationship between the number of people in mental hospitals and prisons, the question regarding whether trans-institutionalisation has replaced de-institutionalisation has become relevant again. Today, there are more institutional options than prisons, and it is important to observe if one of the incentives for the reform of psychiatry, increased integration into society, in principle, has been abandoned and mental hospitals merely been replaced with other forms of institutions such as different forms of forensic facilities and supported housing.

It is also important to follow the development of psychiatric care and interventions from social services. According to Chow and Priebe (2016), the reduction of care and treatment delivered by psychiatry, in the western European countries, continues, while efforts from the municipal social services are increasing. There is an assumption, in the same way as in the case of trans-institutionalisation, of an inverse relationship between contributions from psychiatry and social services, where one effort from one organisation is replaced by interventions from another organisation, which could be called "care and support transfer". In other words, there is an expectation that people with SMI are always in need of care and support, and the only difference is which organisation is conveying it.

The most prominent finding in the study was the successive reductions in psychiatric in- and out-patient care, while social services were increasing. This finding supports the assumption by Chow and Priebe (2016) that the de-institutional process is ongoing. This change is in line with the intentions of the 1995 Swedish psychiatry reform. Increased interventions from social services were meant to compensate for reductions in psychiatric care. The result, however, does not answer the question if the increasing social service interventions in this study are an effect of reduced psychiatric care or simply responding to different and new needs of support for people with SMI.

Few people have been sentenced to prison or forensic psychiatric care, and there is no inverse relationship between reduced in-patient care and the number of persons sentenced to prison or forensic psychiatric care. However, the number of people who have been granted support in housing has increased, especially among men. If this is an alternative to, or has replaced, psychiatric in-patient care, can only be speculated. But the fact that the persons in this study have aged, and the number of people over the age of 65 increased from 152 to 254 people can, at least partly, explain why the proportion of people who have been granted supported housing has increased.

An important and often forgotten factor in the study of people with SMI is economic issues. The women in this study increased their incomes by 6%, men by 4.5%. This should be compared with the Swedish population as a whole, where income increased by 26%. The difference in disposable income between the survey group and the population as a whole has increased from 43% in 2004 to 63% in 2013.

We can speculate that support in housing, employment, etc. has improved the situation for people with SMI but that they face new and other challenges in the form of poverty, which create social exclusion and become an obstacle to achieving the psychiatric reform's goal of becoming a part of society like everyone else.

The notion that persons with a psychosis diagnosis have a life-long need of psychiatric treatment and support from the social services prevails; nonetheless, our results indicate otherwise. In the institutional era, people with SMI often had an ongoing confinement at the mental hospitals and there were few alternatives to in-patient care. The downsizing of the mental hospitals and the re-organisation of psychiatric and social care in the communities were, among other purposes, meant to give persons with SMI a possibility to obtain the status as a citizen like others. An interesting question is if people with SMI are still in the net of psychiatric care providers, albeit outside brick and mortar. In interviews with patients regarding experiences of psychiatric care, one frequent answer is that psychiatric organisations never let go (Bülow et al. 2016). Our study showed that around half of the follow-up population had no institutional care during the last five years of the investigated period, and 13% of the men and 15% of the women had no contact at all with either psychiatry or social services. If they had ended up in another care organisation, for example, in elderly care or in private care, we would have information about that through the Swedish central registers. An educated guess is that at least a majority of those who have no contact at all have recovered and are no longer in need of psychiatric care or social services.

## Limitations

The study has some limitations. The results do not give us answers as to whether the organisational changes or the health and social interventions correspond to the investigated person’s need for care, support and help. We also do not know if they are satisfied with these interventions. Another limitation is that some interventions from the social services are open to everyone, without testing for eligibility, and thereby are not recorded in the database. One example is daily activities that are frequently visited by persons with SMI, which is an intervention lacking information.

## Conclusion

The results show that the de-institutionalisation process is still ongoing with reduced psychiatric care and increasing interventions from social services. At the same time, there is a growing number of persons leaving the systems of care and interventions. There is little likelihood that people could fall between the cracks for five consecutive years, and the same goes for two years, if there was a continued need for care and support. What we cannot control is whether public services have been replaced by interventions provided by family members or close relatives, different types of voluntary organisations or church communities.

There are few differences among men and women. Throughout the studied period, more women were married, had higher education and better financial situation compared to the men. Women were also less likely to live in supported housing or be sentenced to prison or forensic psychiatry. These are factors that could indicate a more favourable situation for the women in the study. However, these differences were not significant.

From the aspect of decreasing psychiatric care and increasing interventions from social services, the purpose of psychiatric reform has been fulfilled. We cannot comment on whether these changes meet the needs of people with SMI or if it is of good quality.

Considering the relative poverty that creates social exclusion and is an obstacle to achieving the goal of psychiatric reform, it is perhaps time to change focus and discuss and study the welfare of people with SMI and expand efforts to combat poverty within the group.
